# A Support vector machine-based mixture cure model for mixed case interval censored data

**DOI:** 10.1007/s11222-025-10796-3

**Published:** 2026-01-16

**Authors:** Suvra Pal, Wisdom Aselisewine

**Affiliations:** 1 Department of Mathematics, University of Texas at Arlington, Arlington, Texas 76019 USA; 2 Division of Data Science, College of Science, University of Texas at Arlington, Arlington, USA

**Keywords:** Machine learning, Platt Scaling, Predictive Accuracy, EM Algorithm, Cure Rate

## Abstract

We propose a semi-parametric two-component model for the analysis of mixed case interval censored (MCIC) data with a cured subgroup. Such data occurs when the time to an event of interest is only known to belong to an interval obtained from a sequence of, say, *k* random examination time points with *k* representing an integer. Furthermore, there is a proportion of subjects who would never be susceptible to the event. The first component of the proposed model describes the probability of cure, and it replaces the traditional generalized linear model with a more flexible support vector machine (SVM)-based approach capable of capturing complex covariate effects. The second component of the proposed model describes the survival distribution of the uncured and is modeled using a Cox proportional hazards structure to preserve the easy interpretation of covariate effects. To the best of our knowledge, this is the first work that employs a machine learning algorithm to analyze MCIC data in the presence of a cured subgroup. To estimate the model parameters, we develop an expectation maximization algorithm. A detailed simulation study demonstrates the superiority of the proposed SVM-based model. Finally, we analyze NASA’s Hypobaric Decompression Sickness Data using the proposed approach.

**Supplementary Information:**

The online version contains supplementary material available at 10.1007/s11222-025-10796-3.

## Introduction

Interval censored data naturally emerge in biological, medical, and demographic studies involving longitudinal follow-up, particularly when dealing with heterogeneous subject populations. In such data, the time to an event of interest cannot be directly observed but is instead confined to an interval determined by a sequence of examination time points (censoring). One approach to characterizing interval censored data is by considering the number of random censoring time points, denoted as *k*. Depending on the value of *k*, three types of interval censored data can be distinguished: Case I interval censored data when $k=1$, often referred to as current status data (Huang 1996; Ma and Kosorok 2005); case *k* interval censored data when $k\ge 2$ (Groeneboom and Wellner 1992); and mixed case interval censored data when *k* is a random integer rather than a fixed number (Schick and Yu 2000; Sen and Banerjee 2007). It is worth noting that with $k>1$, case *k* interval censoring might not be practical, as once it is known that a subject experienced the event by examination time $k^{\prime } (< k)$, further examinations become redundant. Therefore, a more realistic scenario is mixed case interval censoring, where each subject is case *k* interval censored.

A motivating dataset, known as NASA’s Hypobaric Decompression Sickness Data (referred to hereafter as HDSD data), originates from an experiment carried out by NASA focusing on decompression sickness (Conkin et al. 1992). The presence of gas bubbles in venous blood correlates with an elevated risk of decompression sickness in hypobaric environments. A significant level of venous gas emboli (VGE) can precede severe decompression sickness. Therefore, there is considerable interest in modeling the time it takes for grade IV VGE to develop, aiming to anticipate the circumstances in which it is most likely to manifest. The dataset comprises records from subjects who volunteered for denitrogenation test procedures before exposure to a hypobaric environment. Each test involved decompression, with subjects pre-breathing 100% oxygen at site pressure prior to chamber altitude exposure. For each subject, the time until grade IV VGE onset and various covariate values were recorded. The onset time, if it occurred, was noted solely within a time interval. During the experiment, multiple examination times were possible for a subject. In essence, the onset time is case *k* interval censored, where *k* may differ among subjects. Hence, the HDSD data constitutes a mixed case interval censoring scenario.

**Fig. 1:**
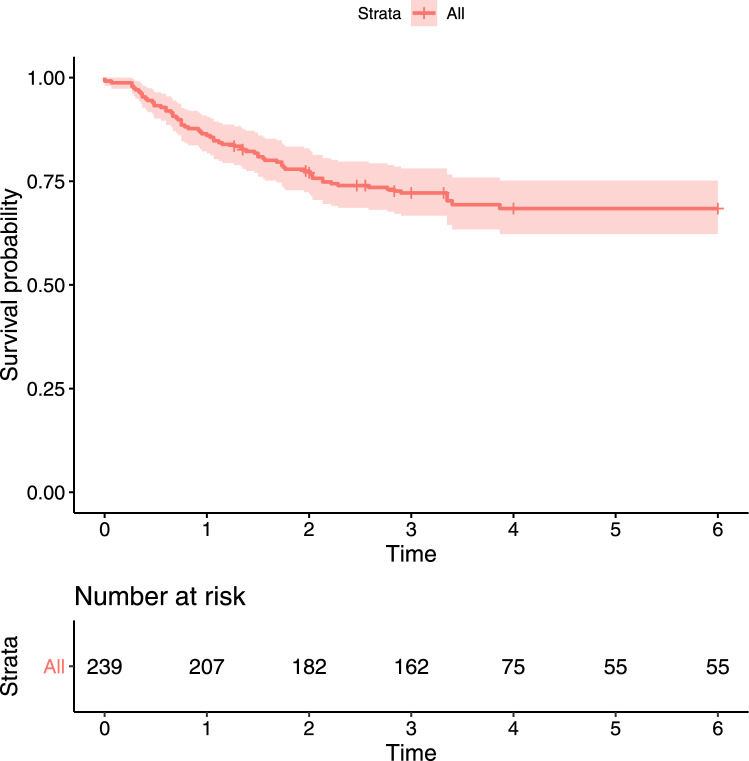
Plot of the NPMLE of the survival function for the HDSD data

A graph depicting the non-parametric maximum likelihood estimate (NPMLE) of the survival function (refer to Figure 1) vividly illustrates a prolonged plateau that eventually stabilizes at a notable non-zero proportion. This observation suggests that certain subjects will never encounter grade IV VGE, regardless of the duration of their stay in the hypobaric chamber. These subjects are commonly referred to as "cured" (Balakrishnan and Pal 2013, 2016; Pal 2021; Wang and Pal 2022). Consequently, the subject population exhibits heterogeneity, comprising a mixture of cured and uncured (or susceptible) individuals. Thus, in addition to addressing the complexities arising from interval censoring, it becomes essential to account for this mixed population. A mixture cure rate model (MCM) enables us to capture this heterogeneity effectively and is therefore justified (Peng and Yu 2021).

For right censored data, the literature on MCM is extensive. For example, Farewell (1982) examined a fully parametric MCM by utilizing logistic regression to model the probability of cure, also known as incidence, and a Weibull distribution to represent the survival distribution of uncured subjects, also referred to as latency. Semiparametric MCMs incorporating proportional hazards (PH) structures for latency have been thoroughly examined by Kuk and Chen (1992), Peng and Dear (2000), and Sy and Taylor (2000), among others. Generalizations to semiparametric proportional odds (Gu et al. 2011), accelerated failure time (Zhang and Peng 2009), and additive hazards (Barui and Yi 2020) within the framework of MCM have also been explored, incorporating various estimation techniques and modeling assumptions. In recent times, there has been a growing focus among researchers on employing machine learning (ML) algorithms for modeling incidence, rather than relying solely on logistic regression. The aim is to capture complex covariate effects, thereby enhancing the predictive accuracy of cure (Li et al. 2020; Xie and Yu 2021; Aselisewine and Pal 2023, 2024, 2025; Aselisewine et al. 2025).

Only a few researchers have delved into the investigation of MCMs with mixed case interval censored data (MCIC). For example, Ma (2010) examined MCIC data by employing a two-component MCM. The first component, concerning incidence, was modeled using parametric logistic regression, while the second component, concerning latency, utilized a Cox’s PH structure. The author explored semi-parametric maximum likelihood estimation (SPMLE) through an efficient computational algorithm. Shao et al. (2014) explored non-parametric smoothing for regression analysis of MCM with MCIC data. Specifically, they investigated varying coefficients and analyzed the consistency and asymptotic distribution of model parameter estimators. They employed a logistic-type model for modeling incidence and a location-scale family of distributions for modeling latency. Zhou et al. (2016) applied MCM to MCIC data, similar to Ma (2010), but introduced a multiple imputation method for estimating model parameters. Chen et al. (2019) examined an MCM for MCIC data, where incidence was modeled using logistic regression and latency was modeled using a semi-parametric transformation model. They developed SPMLE using an expectation maximization (EM) algorithm. For additional literature on interval censored data with a cured subgroup, interested readers can refer to the works of Kim and Jhun (2008), Ma (2009), Xiang et al. (2011), Pal and Balakrishnan (2017a), Treszoks and Pal (2024, 2023, 2025), and Pal and Barui (2024), among others.

The existing literature on analyzing MCIC data using MCM highlights a prevalent trend: the probability of cure (i.e., the incidence) is predominantly addressed through logistic regression or logistic-type models. These models implicitly assume a linear boundary between cured and uncured subjects. However, in the analysis of MCIC data with a cured subgroup, the cured status remains unknown for subjects whose lifetimes are right censored. Therefore, the assumption of a linear classification boundary cannot be validated. Consequently, employing a logistic regression model raises concerns as it might yield biased or imprecise inference for the incidence part, particularly if the true classification boundary is non-linear. Moreover, this imprecision can also affect inference related to latency and overall survival probability. Thus, for analyzing MCIC data with a cured subgroup, we propose a novel MCM. In particular, we model the incidence using a support vector machine (SVM)-based approach, offering greater flexibility to capture complex or non-linear classification boundaries between cured and uncured subjects. To maintain the interpretability of covariate effects on the survival distribution of uncured subjects, we model latency using the Cox PH structure without assuming any specific form for the baseline hazard function. We term our proposed model as MCM-MCIC-SVM. To the best of our knowledge, this is the first endeavor integrating a ML algorithm into the study of MCIC data with a cured subgroup. It is worth noting that the proposed model shares similarities with the ones presented in Pal et al. (2024) and Pal et al. (2023). However, in these works the interval censoring scheme differs from the MCIC scheme considered in this paper. Specifically, the authors did not address the possibility of left-censored data within the interval censoring scheme they investigated, resulting in a likelihood structure that is completely different from what we encounter in this work.

Given the array of ML algorithms at our disposal, we advocate for the utilization of an SVM-based approach in this study primarily because SVM leverages the kernel trick, allowing for the design or fusion of kernels to enhance performance (Pal and Aselisewine 2023). Moreover, SVM’s utilization of a subset of training points in the decision function renders it memory-efficient. Additionally, SVM is anticipated to exhibit shorter execution times compared to alternative classifiers like artificial neural networks. For parameter estimation, we devise an EM algorithm (Pal 2025) employing the sequential minimal optimization (SMO) technique and Platt scaling method (Platt 1999b). We compare our proposed MCM-MCIC-SVM model with the logistic regression-based MCM model (MCM-MCIC-Logit) and the spline-based MCM model (MCM-MCIC-Spline), acknowledging that spline-based models can also capture complex or non-linear data patterns (Chen and Du 2018). We demonstrate that when confronted with non-linear or complex classification boundaries, the proposed MCM-MCIC-SVM model yields more precise (i.e., reduced bias) and efficient (i.e., decreased mean square error) estimates of cured/uncured probabilities in comparison to MCM-MCIC-Logit and MCM-MCIC-Spline models. Additionally, we illustrate that our proposed model attains the highest predictive accuracy for cure. Furthermore, we establish that our model’s capacity to capture complex classification boundaries enhances estimation outcomes concerning the latency component and overall population survival.

The rest of this paper is organized as follows. Section 2 outlines the formulation of the proposed MCM-MCIC-SVM model. Section 3 details the development of the estimation method utilizing the EM algorithm. Section 4 showcases the outcomes of an extensive simulation study, illustrating the performance and superiority of the proposed model across various scenarios. In Section 5, we provide an application by analyzing the HDSD data. Finally, Section 6 offers concluding remarks and identifies future research directions that integrate machine learning with cure rate models.

## Form of data and proposed model

Consider *T* as the true time to an event of interest. Within the framework of MCIC, censoring is determined through a two-step procedure (Ma 2010). Firstly, the count of random censoring times, denoted as *k*, is established. Subsequently, the observation is defined by a case *k* interval censoring scheme. Let $(U_1,U_2,\cdots ,U_k)$ represent the *k* random censoring times. It is important to note that only $(U_L,U_R)$ holds relevance in constructing the likelihood structure, where $(U_L,U_R)$ denotes the shortest interval satisfying $U_L<T\le U_R$. Additionally, we account for the potential scenarios of $U_L=0$ (left censoring) or $U_R=\infty$ (right censoring). For clarity in notation, let the left censoring and interval censoring indicators be respectively defined as: $\delta _1=I(U_L=0)$ and $\delta _2=I(U_L>0 \ \ \mathrm{ \& } \ \ U_R < \infty )$, where $I(\cdot )$ denotes the indicator function. Moreover, we assume $P(U_1=0)=0$, implying that the first examination time occurs only after the study commencement, a reasonable assumption in biomedical studies. To accommodate the possibility of cure, we introduce a cured status variable *J*, defined as: $J=0$ if a subject is cured or immune to the event of interest (i.e., $T=\infty$) and $J=1$ otherwise. It is worth noting that *J* assumes the value 1 for left-censored and interval-censored observations, while *J* remains unknown for all right-censored observations. Let $\boldsymbol{z}$ denote a vector of covariates linked with the incidence, and $\boldsymbol{x}$ represent another vector of covariates associated with the latency. Importantly, $\boldsymbol{x}$ and $\boldsymbol{z}$ may share common elements. Consequently, the observed data is represented as $D_O=\{U_{Li},U_{Ri},\delta _{1i},\delta _{2i},\boldsymbol{x_i},\boldsymbol{z_i}\}, i=1,2,\cdots ,n$, with *n* denoting the sample size.

### Proposed SVM-based model

The aforementioned data structure is represented through a two-component mixture model. The first component models the probability of cure (incidence), while the second component models the survival distribution of the uncured subjects (latency). Such a two-component model can be characterized by its conditional survival function, expressed as follows:

1 $$\begin{aligned} S_p(t|\boldsymbol{x},\boldsymbol{z}) = 1-\pi (\boldsymbol{z}) + \pi (\boldsymbol{z}) S(t|\boldsymbol{x}). \end{aligned}$$

In equation (1), $1-\pi (\boldsymbol{z})$ denotes the probability of cure (or cure rate), and $S(t|\boldsymbol{x})$ represents the conditional survival function of the uncured subjects. It is important to note that $S_p(t|\boldsymbol{x},\boldsymbol{z})$, representing the overall population survival function, is improper, given that $S_p(\infty |\boldsymbol{x},\boldsymbol{z}) = 1-\pi (\boldsymbol{z}) (>0)$. We propose modeling $\pi (\boldsymbol{z})$ using an SVM-based approach, diverging from the conventional approach involving a generalized linear model with a logit link function. For modeling $S(t|\boldsymbol{x})$, we adopt a Cox PH structure, leading to the following expression:

2 $$\begin{aligned} S(t|\boldsymbol{x}) = \{S_0(t)\}^{\exp (\boldsymbol{x}^\prime \boldsymbol{\beta })}. \end{aligned}$$

In eqn.(2), $S_0(t)$ represents the common baseline survival function of the uncured subjects, and $\boldsymbol{\beta }$ represents the vector of regression parameters (excluding an intercept) associated with $\boldsymbol{x}$. In this paper, we suggest estimating $S_0(\cdot )$ utilizing the non-parametric Turnbull estimator (Turnbull 1976), thereby avoiding any parametric distributional assumptions. This estimator is devoid of a closed form and is constructed through an iterative procedure. For further insights into this iterative approach, interested readers can refer to Pal et al. (2023).

### Modeling cure/uncure probability with SVM

We propose an innovative modeling approach for $\pi (\boldsymbol{z})$ using SVM with the aim of capturing non-linearity within the data. SVM, a supervised machine learning algorithm, seeks a hyperplane in a multidimensional feature space to maximize the margin or separating space between two classes (Cortes and Vapnik 1995). In our context, the two classes correspond to cured and uncured groups. SVM offers appeal in its ability to handle complex, inseparable data by transforming it into a higher-dimensional space using a kernel trick. Consequently, SVM is regarded as a robust and flexible classifier compared to traditional logistic, probit, or complementary log-log link functions. Given a set of covariates $\boldsymbol{z}_i$ (associated with the *i*-th subject) and assuming known cured/uncured statuses for all subjects, SVM endeavors to construct an optimal decision or classification rule described as:

3 $$\begin{aligned} g(\boldsymbol{z})=\sum _{i=1}^n c_i V_i K(\boldsymbol{z}_i, \boldsymbol{z}) - b, \end{aligned}$$

where $V_i=1$ if the *i*-th subject is uncured (or susceptible) and $V_i=-1$ if the *i*-th subject is cured. Here, $K(\cdot ,\cdot )$ denotes a suitable kernel function, and $c_i$’s and *b* are unknown parameters. The kernel function $K(\cdot ,\cdot )$ is typically chosen to be a symmetric positive semi-definite function. In our case, we opt for a radial basis kernel function, given by:

4 $$\begin{aligned} K(\boldsymbol{z}_i, \boldsymbol{z}_j)=\exp \left\{ -\frac{(\boldsymbol{z}_i-\boldsymbol{z}_j)^{\prime }(\boldsymbol{z}_i-\boldsymbol{z}_j)}{2\sigma ^2}\right\} , \end{aligned}$$

where the parameter $\sigma$ determines the kernel width. Alternatively, one may use other kernel functions such as polynomial or sigmoid kernel functions. The derivation of (3) begins with the primal SVM formulation. Let $\phi (\cdot )$ be a feature mapping into a higher-dimensional space. The primal optimization problem is:

5 $$\begin{aligned} \min _{\boldsymbol{w}, b, \boldsymbol{\xi }} \ \frac{1}{2}\Vert \boldsymbol{w}\Vert ^2 + Q \sum _{i=1}^n \xi _i, \end{aligned}$$

subject to

6 $$\begin{aligned} V_i \big (\boldsymbol{w}^{\prime }\phi (\boldsymbol{z}_i) + b\big ) \ge 1 - \xi _i, \quad \xi _i \ge 0, \quad i=1,\dots ,n. \end{aligned}$$

Here, $\xi _i$ are slack variables that allow misclassification, while $Q>0$ controls the trade-off between maximizing the margin and penalizing classification errors. By applying the method of Lagrange multipliers, one obtains the dual quadratic programming problem:

7 $$\begin{aligned} \underset{c_1, \dots , c_n}{\max }\left[ -\frac{1}{2} \sum _{i=1}^n \sum _{j=1}^n c_i c_j V_i V_j K(\boldsymbol{z}_i, \boldsymbol{z}_j) + \sum _{i=1}^n c_i \right] , \end{aligned}$$

subject to

8 $$\begin{aligned} \sum _{i=1}^n c_i V_i=0, \qquad 0\le c_i \le Q, \quad i=1, \dots , n. \end{aligned}$$

Thus, the misclassification penalty parameter *Q* from the primal formulation appears naturally as an upper bound on the Lagrange multipliers $c_i$ in the dual problem. To solve the dual optimization problem in (7), we employ the sequential minimal optimization (SMO) method (Platt 1999a), preferred for its ability to decompose a large quadratic programming problem into smaller subproblems. SMO is well-suited for handling very large training datasets and is computationally efficient. Once the parameters $c_i$ are estimated, the threshold *b* can be determined by:

9 $$\begin{aligned} b=\sum _{i=1}^n c_i V_i K(\boldsymbol{z}_i, \boldsymbol{z}_{j})-V_j, \end{aligned}$$

for some $c_j > 0$. For a subject with covariate vector $\boldsymbol{z}$, SVM classifies the subject as belonging to the uncured group if $g(\boldsymbol{z})>0$, and as belonging to the cured group if $g(\boldsymbol{z})<0$. Both parameters *Q* and $\sigma$ are tuned through cross-validation to achieve optimal classification accuracy.

In addition to classifying subjects into cured or uncured groups, it is also crucial to estimate the probabilities of being cured or uncured. For this purpose, we employ the Platt scaling method (Platt 1999b), which fits a sigmoid to map SVM outputs to posterior probabilities. Using the Platt scaling method, the estimates of posterior probabilities are given by:

10 $$\begin{aligned} {\pi (\boldsymbol{z})} = \frac{1}{1+\exp \{A g(\boldsymbol{z})+ B\}}, \end{aligned}$$

where *A* and *B* are solutions to the following maximization problem:

11 $$\begin{aligned} \max _{A,B}\bigg [\sum _{i=1}^n (1- \tilde{n}_i)[A g(\boldsymbol{z}_i) + B] - \sum _{i=1}^n\log [1 + \exp \{A g(\boldsymbol{z}_i) + B\}]\bigg ]. \end{aligned}$$

In eqn.(11), $\tilde{n}_i$ defined as:

12 $$\begin{aligned} \tilde{n}_i = {\left\{ \begin{array}{ll} \frac{n^{(1)}+1}{n^{(1)}+2}, & \text { if } V_i=1\\ \frac{1}{n^{(0)}+2}, & \text { if } V_i=-1, \end{array}\right. } \end{aligned}$$

where $n^{(1)}$ is the number of uncured subjects and $n^{(0)}$ is the number of cured subjects, with $n=n^{(1)}+n^{(0)}$.

### Tuning the proposed SVM-based model

We tackle the challenge of overfitting or underfitting through two strategies. Firstly, we divide the data into two distinct sets: the training set and the testing set. The training set is employed to train the proposed model, while the testing set is utilized to assess or validate the predictive accuracy of the finalized model. Secondly, we conduct grid-search cross-validation tuning to explore the optimal values of the two most crucial hyperparameters of our model: the cost parameter (referred to as *Q*) and the parameter $\gamma$, where $\gamma = \frac{1}{2\sigma ^2}$, during the training phase of the model. The parameter *Q* acts as a penalization parameter for misclassification within the fitted model. A higher value of *Q* implies a more significant penalty for misclassification, and vice versa. On the other hand, $\gamma$ governs the impact of similarity of a training point, which in turn affects the model’s performance. A higher value of $\gamma$ indicates a smaller similarity radius, and vice versa. Through grid-search cross-validation, we define various plausible values for each hyperparameter where, $\gamma = { 2^{-6},2^{-5},2^{-4}}$ and $Q = { 2^{4},2^5,2^6}$. To select optimal hyperparameters, we performed grid search with 10-fold cross-validation. For each candidate pair $(\gamma , Q)$ in the search grid, the misclassification error rate obtained from the cross-validation was used as a tuning criterion or metric. The parameter set that achieved the lowest cross-validated misclassification error rate was selected for model fitting. Subsequently, the final fitted model was evaluated on an independent test set, where the area under the graphical receiver operating characteristic (ROC) curve (AUC) was used as the primary measure to gauge the effectiveness of the final model. This separation ensures that hyperparameter selection was based on the cross-validation misclassification error rate, while the AUC was reserved for final model evaluation.

It is also possible to adopt a data-driven approach to setting the kernel width parameter $\gamma$. Specifically, we compute a baseline value

$$ \gamma _0 = \frac{1}{\operatorname {median}\{\Vert \textbf{z}_i - \textbf{z}_j\Vert ^2 : i<j\}}, $$

i.e., the reciprocal of the median squared pairwise distance among training points. This ensures that the kernel reflects the intrinsic scale of the covariates. To allow flexibility around this data-adaptive method, we construct a multiplicative grid

$$ \gamma = \left\{ \gamma _0 \times 2^k \; : \; k = -3, -2, -1, 0, 1, 2, 3 \right\} , $$

and select the optimal value via 10-fold cross-validation. Note that the grid of *Q* values can remain unchanged (i.e., $2^4, 2^5, 2^6$), or we may extend the grid to include a wider range such as $Q = \{2^{-3}, 2^{-2}, 2^{-1}, 2^0, 2^1, 2^2, 2^3,2^4,2^5,2^6\}$. This strategy directly ties the choice of $\gamma$ to the variability in the data, while still allowing for fine-tuning through cross-validation.

## Likelihood function and EM algorithm

Within the framework of the MCIC mechanism, and considering the observed data $D_O$, the log-likelihood function for the observed data can be formulated as follows:

13 $$\begin{aligned} l_o= &  \sum _{i=1}^n [\delta _{1i}\{ \log (\pi (\boldsymbol{z}_i)) + \log (1-S_0(U_{Ri})^{\exp (\boldsymbol{x}_i^\prime \boldsymbol{\beta })}) \} ] \nonumber \\ &  + \sum _{i=1}^n [\delta _{2i}\{ \log (\pi (\boldsymbol{z}_i)) + \log (S_0(U_{Li})^{\exp (\boldsymbol{x}_i^\prime \boldsymbol{\beta })}\nonumber \\ &  -S_0(U_{Ri})^{\exp (\boldsymbol{x}_i^\prime \boldsymbol{\beta })}) \} ] \nonumber \\ &  + \sum _{i=1}^n [ (1-\delta _{1i}-\delta _{2i}) \log \{1-\pi (\boldsymbol{z}_i) \nonumber \\ &  + \pi (\boldsymbol{z}_i)S_0(U_{Li})^{\exp (\boldsymbol{x}_i^\prime \boldsymbol{\beta })} \} ]. \end{aligned}$$

Given that the cured statuses, $J_i$, are unknown for all right-censored observations, the complete dataset is denoted as $D_C=\{U_{Li},U_{Ri},\delta _{1i},\delta _{2i},J_i,\boldsymbol{x}_i,\boldsymbol{z}_i\}$ for $i=1,2,\cdots ,n$, encompassing both observed and missing $J_i$’s. Subsequently, the complete data log-likelihood function, after some algebra, can be expressed as:

14 $$\begin{aligned} l_c = l_{c1} + l_{c2}, \end{aligned}$$

where

15 $$\begin{aligned} l_{c1}= &  \sum _{i=1}^n [ \delta _{1i}\log (\pi (\boldsymbol{z}_i)) + \delta _{2i}\log (\pi (\boldsymbol{z}_i))] \nonumber \\ &  + \sum _{i=1}^n (1-\delta _{1i}-\delta _{2i}) \{ (1-J_i)\nonumber \\ &  \log (1-\pi (\boldsymbol{z}_i)) + J_i \log (\pi (\boldsymbol{z}_i)) \} \end{aligned}$$

and

16 $$\begin{aligned} l_{c2}= &  \sum _{i=1}^n [\delta _{1i} \log \{1-S_0(U_{Ri})^{\exp (\boldsymbol{x}_i^\prime \boldsymbol{\beta })}\} + \delta _{2i} \log \{S_0(U_{Li})^{\exp (\boldsymbol{x}_i^\prime \boldsymbol{\beta })} \nonumber \\ &  - S_0(U_{Ri})^{\exp (\boldsymbol{x}_i^\prime \boldsymbol{\beta })}\} ] \nonumber \\ &  + \sum _{i=1}^n (1-\delta _{1i}-\delta _{2i}) \log \{S_0(U_{Li})^{\exp (\boldsymbol{x}_i^\prime \boldsymbol{\beta })+\log (J_i)}\}. \end{aligned}$$

Note that $l_{c1}$ is a function related to the incidence only whereas $l_{c2}$ is a function related to latency only.

At the $(r+1)^{th}$ step of the EM algorithm, the expectation step (E-step) computes the conditional expectation of $l_c$, given the observed data $D_O$ and the current estimates of model parameters, i.e., $\boldsymbol{\theta }^{(r)} = (\pi ^{(r)}(\boldsymbol{z}_i),\hat{S_0}(\cdot ),\boldsymbol{\beta }^{(r)})$ for $i=1,2,\cdots ,n$, where $\hat{S_0}(\cdot )$ is the Turnbull estimate of $S_0(\cdot )$ and remains fixed across EM iterations. This reduces to computing the following conditional expectation of $J_i$ for $i=1,2,\cdots ,n$:

17 $$\begin{aligned} w_i^{(r+1)}= &  P[J_i = 1 | D_O,\boldsymbol{\theta }^{(r)}] \nonumber \\= &  \delta _{1i} + \delta _{2i} + (1-\delta _{1i}-\delta _{2i}) \nonumber \\ &  \frac{\pi ^{(r)}(\boldsymbol{z}_i)\hat{S_0}(U_{Li})^{\exp (\boldsymbol{x}_i^\prime \boldsymbol{\beta }^{(r)})}}{1-\pi ^{(r)}(\boldsymbol{z}_i) + \pi ^{(r)}(\boldsymbol{z}_i)\hat{S_0}(U_{Li})^{\exp (\boldsymbol{x}_i^\prime \boldsymbol{\beta }^{(r)})}}. \end{aligned}$$

Thus, at the $(r+1)^{th}$ step of the EM algorithm, the E-step replaces the $J_i$’s in $l_{c1}$ and $l_{c2}$ with $w_i^{(r+1)}$. Let us denote the resulting functions by $Q_{c1}$ and $Q_{c2}$.

In the maximization step, or M-step, of the EM algorithm, the conventional approach in this context is to separately maximize $Q_{c1}$ and $Q_{c2}$ to derive updated estimates of $\pi (\boldsymbol{z}_i)$ and $\boldsymbol{\beta }$, respectively. However, in this study, we deviate from maximizing the function $Q_1$ to estimate $\pi (\boldsymbol{z}_i)$ after presuming a parametric form for $\pi (\boldsymbol{z}_i)$. Instead, as explained in Section 2.2, we utilize SVM to initially derive the classification rule $g(\boldsymbol{z}_i)$ (as expressed in eqn.(3)), followed by employing the Platt scaling method (as outlined in eqn.(10)) to estimate the posterior uncured probabilities $\pi (\boldsymbol{z}_i)$. To utilize SVM, it is necessary to have the class label values $V_i$ (or $J_i$) for $i=1,2,\cdots ,n$. However, these values are unknown for all subjects with right-censored lifetimes. To address this challenge, we propose imputing the values of $V_i$ using a multiple imputation-based technique, inspired by Li et al. (2020). To implement this, during the $(r+1)^{th}$ iteration step of the EM algorithm, we utilize the conditional probability of being uncured, as defined in eqn.(17), to generate ${ J_i^{(s)}, i\in \Delta _0;s=1,2,\cdots ,M }$ from a Bernoulli distribution with a success probability of $w_i^{(r+1)}$, where *M* is a large positive integer and $\Delta _0$ denotes the set of right censored observations. If $J_i^{(s)}=1$, we assign $V_i^{(s)}=1$; otherwise, we assign $V_i^{(s)}=-1$. Typically, the value of *M* is chosen as 5 for practical purposes (Li et al. 2020). After generating $V_i^{(s)}, i\in \Delta _0$, for each $s=1,2,\cdots ,M$, we estimate $\pi (\boldsymbol{z}_i)$ by employing SVM followed by the Platt scaling method. These estimates are denoted as $\pi _s^{(r+1)}(\boldsymbol{z}_i)$. Ultimately, for each $i=1,2,\cdots ,n$, we derive the estimate of $\pi (\boldsymbol{z}_i)$ as: $\pi ^{(r+1)}(\boldsymbol{z}_i)=\frac{1}{M}\sum _{s=1}^M\pi _s^{(r+1)}(\boldsymbol{z}_i)$.

To update the estimate of $\boldsymbol{\beta }$, we aim to maximize the function $Q_{c2}$ with respect to $\boldsymbol{\beta }$, which entails finding:

18 $$\begin{aligned} \boldsymbol{\beta }^{(r+1)}=\underset{\boldsymbol{\beta }}{\arg \max }\text  Q_{c2}. \end{aligned}$$

The maximization outlined in equation (18) can be executed using the “optim()" function within the R software, specifying the method as “Nelder-Mead". Additionally, for alternative optimization methods, one may explore new approaches based on the non-linear conjugate gradient algorithm incorporating an efficient line search technique, as discussed in works by Pal and Roy (2021, 2022, 2023). The E-step and the M-step are iteratively conducted until a convergence criterion is met, typically defined as:

19 $$\begin{aligned} || \boldsymbol{\theta }^{(r+1)} - \boldsymbol{\theta }^{(r)} ||^2_2 < \epsilon , \end{aligned}$$

where $\epsilon$ represents a specified tolerance level (e.g., $10^{-3}$ or $10^{-5}$), and $||\cdot ||_2$ signifies the $L_2$-norm. Given the complexity of the proposed EM algorithm, the standard errors of the estimates can be obtained using a bootstrap procedure, as outlined in the works of Peng and Dear (2000) and Li et al. (2020).

To initiate the iterative EM algorithm, initial guesses for $\pi (\boldsymbol{z}_i)$ and $\boldsymbol{\beta }$, for $i=1,2,\cdots ,n$, are required. Firstly, for an initial estimate of $\pi (\boldsymbol{z}_i)$, we use $\delta _{1i}+\delta _{2i}$ as an indicator for the cured status $V_i$. Consequently, if $\delta _{1i}+\delta _{2i}=1$, we assign $V_i=1$, and if $\delta _{1i}+\delta _{2i}=0$, we assign $V_i=-1$. Subsequently, we employ the SVM followed by the Platt scaling method to obtain $\pi (\boldsymbol{z}_i)$. Secondly, for an initial estimate of $\boldsymbol{\beta }$, we utilize the observed data log-likelihood function excluding any cure probability and assume a Cox’s PH structure to estimate $\boldsymbol{\beta }$. This procedure can be conveniently executed using the R function “ic_sp()" from the package “icenReg".

## Simulation study

We conduct an extensive Monte Carlo simulation study to illustrate the performance of both the proposed model and the EM algorithm. Additionally, we compare the performance of our proposed model with that of logit-based and spline-based models. Performance metrics encompass biases and mean square errors (MSEs) of various quantities of interest, such as the uncured probability, susceptible survival probability, and overall survival probability, along with the predictive accuracies for cure using the AUC values. To fit the spline-based model, we employ a non-parametric additive model with a thin plate spline. We utilize the “gam()" function from the R package “mgcv", which automatically selects the effective degrees of freedom for each covariate function. Our simulations consider two different sample sizes: $n=300$ and $n=600$. We split the data into two-thirds for the training set and one-third for the testing set. We explore the following four scenarios to generate the true uncured probabilities:

$$\begin{aligned} &  \text {Scenario 1:} \ \ \pi (\boldsymbol{z}) = \frac{ \exp (0.3 - 5z_{1} - 3z_{2})}{1 + \exp (0.3 - 5z_{1} - 3z_{2})},\\ &  \text {Scenario 2:} \ \ \pi (\boldsymbol{z}) = \frac{ \exp (0.3 + 5z_{1}z_{2} - {3}z_{1}z_{2})}{1 + \exp (0.3 + 5z_{1}z_{2} - {3}z_{1}z_{2})},\\ &  \text {Scenario 3:} \ \ \pi (\boldsymbol{z})=\exp (-\exp (-0.8z_1z_2+1.1z_2z_4 \\ &  \qquad \qquad \qquad \qquad \qquad +0.5z_3+0.2z_7^{2}-1.3 \text {sin}(z_5z_6)\\ &  \qquad \qquad \qquad \qquad \qquad +1.9 \text {cos}(z_7z_8)-1.5\exp (z_5z_6z_7)\\ &  \qquad \qquad \qquad \qquad \qquad -1.6z_7z_8z_9z_{10} \\ &  \qquad \qquad \qquad \qquad \qquad +0.8z_6z_7z_8^{2}z_9^{2}\\ &  \qquad \qquad \qquad \qquad \qquad +1.8 \text {cos}(z_5z_6z_7z_8z_9)\\ &  \qquad \qquad \qquad \qquad \qquad +1.2\mid (z_6z_7z_8z_9z_{10})\mid ^{0.5}-2.4)),\\ &  \text {Scenario 4:} \ \ \pi (\boldsymbol{z})=\exp (-\exp (-0.8z_1z_2+1.1z_2z_4 \\ &  \qquad \qquad \qquad \qquad \qquad +0.5z_3+0.2z_7^{2}-1.3 \text {sin}(z_5z_6)\\ &  \qquad \qquad \qquad \qquad \qquad +1.9 \text {cos}(z_7z_8)-1.5\exp (z_5z_6z_7)\\ &  \qquad \qquad \qquad \qquad \qquad -1.6z_7z_8z_9z_{10} \\ &  \qquad \qquad \qquad \qquad \qquad +0.8z_6z_7z_8^{2}z_9^{2}\\ &  \qquad \qquad \qquad \qquad \qquad +1.8 \text {cos}(z_5z_6z_7z_8z_9)\\ &  \qquad \qquad \qquad \qquad \qquad +1.2\mid (z_6z_7z_8z_9z_{10})\mid ^{0.5}-2.4)). \end{aligned}$$

**Fig. 2:**
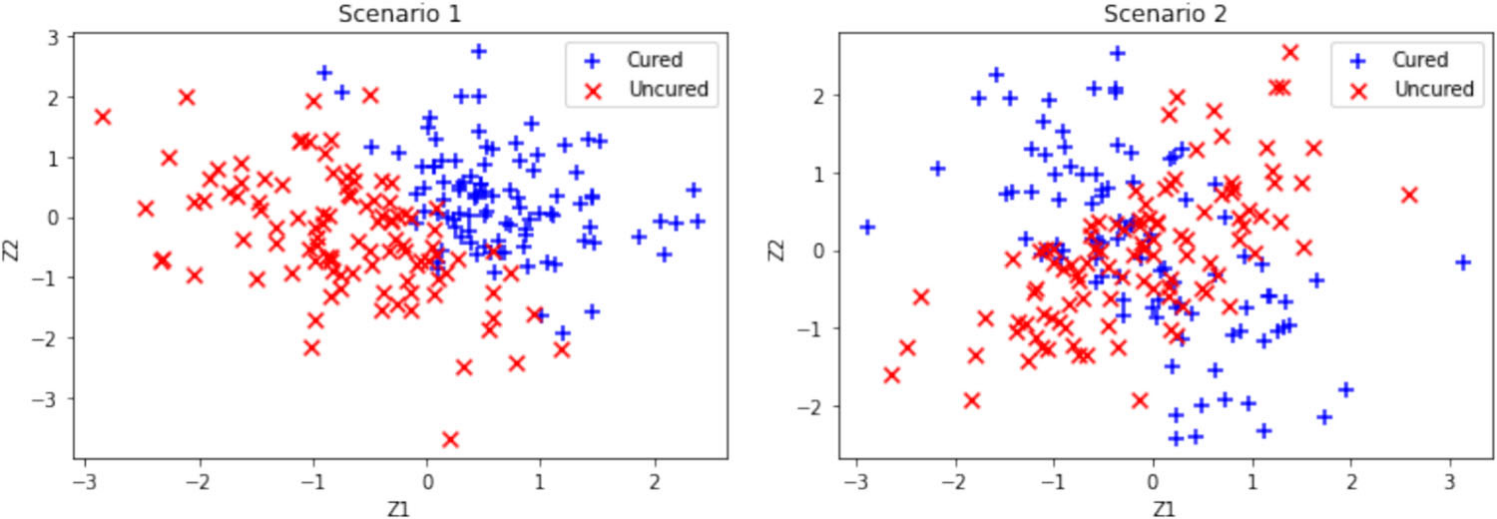
Simulated cured and uncured observations for scenarios 1 and 2

In scenarios 1-2, $z_1$ and $z_2$ are independently generated from a standard normal distribution, and we consider $\boldsymbol{z} = \boldsymbol{x}$. Specifically, scenario 1 illustrates a conventional logistic function, implying a linearly separable boundary for classification between cured and uncured subjects based on covariates. In scenarios 2, interaction terms are introduced into the link function, yielding a non-linear classification boundary; refer to Figure 2. To assess the robustness and versatility of our proposed model across diverse data scenarios, we introduce scenarios 3 and 4. In scenario 3, we generate 10 correlated covariates from a multivariate normal distribution, $N_{10}(0, \Sigma )$, where $\Sigma$ represents the variance-covariance matrix with elements defined by $\sigma _{ij}=0.7^{|i-j|}$, $1 \le i, j \le 10$. In scenario 4, we sample $z_1,z_2,z_3$, and $z_4$ from a Bernoulli distribution with success probabilities of 0.5, 0.3, 0.5, and 0.7, respectively, while $z_5, z_6, \cdots ,z_{10}$ are drawn from a standard normal distribution. Furthermore, in Scenario 4, all 10 covariates are utilized in the latency part, whereas only a subset of 5 covariates is chosen for the incidence part, ensuring $\boldsymbol{z} \ne \boldsymbol{x}$. Conversely, in Scenario 3, the same set of covariates is employed for both incidence and latency modeling. Both scenarios 3 and 4 entail more intricate link functions that involve multiple covariates and complex interaction terms.

For the latency component, we assume a PH structure for the hazard function of uncured subjects, given by

$$\begin{aligned} h(t;\boldsymbol{x}) = h_0(t)e^{\boldsymbol{x}^\prime \boldsymbol{\beta }}, \end{aligned}$$

where the baseline hazard function is specified as $h_0(t)=\alpha t^{\alpha -1}$ with $\alpha >0$. In scenarios 1 and 2, the chosen true values for $(\alpha ,\beta _1,\beta _2)$ are $(1,-5,5)$. For scenarios 3, and 4, we opt for $(\alpha ,\beta _1,\beta _2,\beta _3,\beta _4,\beta _5,\beta _6,\beta _7,\beta _8,\beta _9,\beta _{10})$ as $(0.2,-0.8,1.5,0.5,1.3,-0.6,-1.4,-0.5,-0.8,0.5,1.8)$. With these configurations, the approximate true proportions of cured and censored data for scenarios 1-4 are (0.47, 0.64), (0.43, 0.68), (0.40, 0.78), and (0.60, 0.75), respectively. It is worth noting that with the chosen form for the baseline hazard function, the lifetimes of susceptible individuals follow a Weibull distribution with a shape parameter $\alpha$ and a scale parameter $\{e^{\boldsymbol{x}^\prime \boldsymbol{\beta }}\}^{-1/\alpha }$. To generate mixed case interval censored data $(U_{Li},U_{Ri},\delta _{1i},\delta _{2i}), i=1,2,\cdots ,n$, we carry out the following steps:

- Step 1: Generate $C_i$ from a Uniform (0,1) distribution.
- Step 2: If $C_i\le 1-\pi (\boldsymbol{z}_i)$, set $t_i=\infty$. Otherwise, generate $t_i$ from a Weibull distribution with shape parameter $\alpha$ and scale parameter $\{e^{\boldsymbol{x}_i^\prime \boldsymbol{\beta }}\}^{-1/\alpha }$.
- Step 3: Generate censoring times $U_j=\sum _{l=1}^j \xi _l$, where $\xi _l\sim Uniform(0.1,0.25)$. Keep generating $\xi _l$ until $U_j > \min \{t_i,2.5\}$.
- Step 4: Depending on the value of $t_i$,
  - If $t_i>2.5$, set $U_{Li}=U_j, U_{Ri}=\infty , \delta _{1i}=0$, and $\delta _{2i}=0$.
  - If $t_i\le 0.1$, set $U_{Li}=0, U_{Ri}=U_j, \delta _{1i}=1$, and $\delta _{2i}=0$.
  - If $0.1<t_i\le 2.5$, set $U_{Li}=U_{j-1}, U_{Ri}=U_j, \delta _{1i}=0$, and $\delta _{2i}=1$.

All simulations are performed using the R statistical software, and the results are based on 200 Monte Carlo iterations. To utilize the SVM, we opt for 5 imputations for the multiple imputation-based technique to estimate $\pi (\boldsymbol{z}_i)$, following the approach outlined by Li et al. (2020). The R codes for both data generation and the SVM-based EM algorithm can be found on GitHub; see https://github.com/Aselisewine/SVM-BASED-CURE-MODEL-MIXED-CASE-DATA.

### Simulation results

In Table 1, we present the biases and MSEs of the estimates for uncured probabilities derived from the proposed MCM-MCIC-SVM model, contrasting them with those obtained from the MCM-MCIC-Spline and MCM-MCIC-Logit models. Notably, when non-linear boundaries delineate cured and uncured subjects (as seen in scenarios 2-4), the biases and MSEs of the estimated uncured probabilities from the proposed MCM-MCIC-SVM model are smaller compared to those from the MCM-MCIC-Spline and MCM-MCIC-Logit models. Conversely, in scenarios where the true classification boundary is linear (scenario 1), the MCM-MCIC-Logit model yields the least bias and MSE in the estimated uncured probability. This outcome is expected, given that a logistic regression-based model is more adept at capturing linear classification boundaries. The results from Table 1 unequivocally underscore that the proposed MCM-MCIC-SVM model adeptly captures complex relationships between uncured probabilities and covariates, thereby enhancing the accuracy and precision of uncured probability estimation. Notably, both biases and MSEs decrease as sample size increases.

**Fig. 3:**
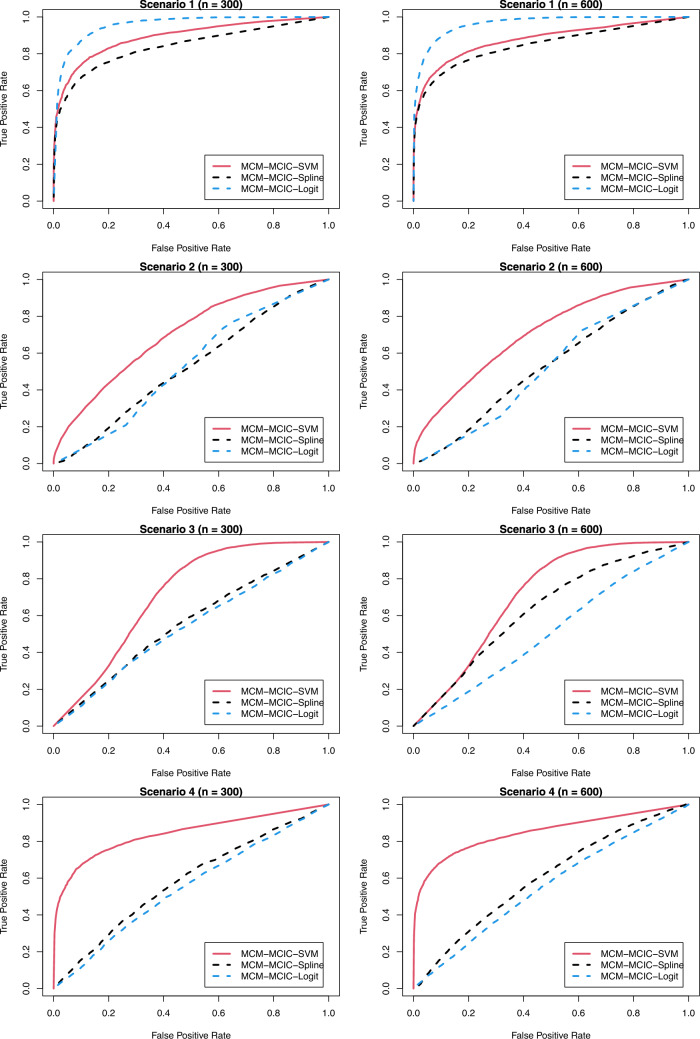
ROC curves for different models

**Table 1:** Comparison of biases and MSEs of the uncured probability for different models

		Bias			MSE		
Scenario	n	SVM	Spline	Logit	SVM	Spline	Logit
1	300	-0.0961	-0.1221	0.0362	0.0827	0.1140	0.0185
	600	-0.0881	-0.1125	0.0334	0.0705	0.0969	0.0128
2	300	-0.0670	0.1246	0.1308	0.0713	0.1154	0.2186
	600	-0.0661	0.1136	0.1083	0.0629	0.1140	0.2028
3	300	0.0654	0.1344	0.1642	0.1151	0.1933	0.1637
	600	0.0625	0.1268	0.1592	0.1121	0.1724	0.1566
4	300	0.0507	0.0837	0.1532	0.1101	0.1536	0.1980
	600	0.0403	0.0819	0.1271	0.1010	0.1268	0.1633

**Table 2:** Comparison of AUC values for different models

Scenario	n	Training AUC			Testing AUC		
		SVM	Spline	Logit	SVM	Spline	Logit
1	300	0.8976	0.8424	0.9683	0.8984	0.8442	0.9654
	600	0.8798	0.8551	0.9693	0.8793	0.8517	0.9688
2	300	0.7220	0.5464	0.5345	0.7122	0.5445	0.5452
	600	0.7160	0.5410	0.5242	0.7112	0.5372	0.5274
3	300	0.7811	0.6479	0.5728	0.7411	0.5701	0.5543
	600	0.7722	0.6293	0.5668	0.7396	0.6194	0.5647
4	300	0.8632	0.6891	0.5772	0.8422	0.5895	0.5553
	600	0.8926	0.6694	0.5785	0.8781	0.6055	0.5547

It is worth noting that in practical settings, the cured statuses remain unknown for all right-censored observations. Nevertheless, in a simulation study, it becomes feasible to ascertain whether a right-censored observation can be classified as cured. Leveraging such insights into the cured statuses, there is a keen interest in comparing the MCM-MCIC-SVM model against the MCM-MCIC-Spline and MCM-MCIC-Logit models by examining ROC curves and the corresponding AUC values to discern which model yields the highest predictive accuracy for cure. In Table 2, we present the AUC values across different scenarios considered, while Figure 3 showcases the ROC curves. The true label for AUC calculation corresponds to the true cure index for each subject during data generation. From the results in Table 2, it becomes evident once more that under complex (non-linear) classification boundaries, the MCM-MCIC-SVM model outperforms the MCM-MCIC-Spline and MCM-MCIC-Logit models, exhibiting noticeable disparities in AUC values. Conversely, under a linear classification boundary, the performance of the MCM-MCIC-SVM model closely aligns with that of the MCM-MCIC-Logit model. The proximity between training and testing AUC values suggests no issues with model overfitting. The findings from Table 2 unequivocally demonstrate that our proposed model’s capability to capture complex classification boundaries leads to significantly improved predictive accuracies for cure.

In Tables 3 and 4, we present a comparative analysis of the biases and MSEs associated with estimates of overall survival probabilities and susceptible survival probabilities, respectively, across different models. Observing Tables 3 and 4, it becomes evident that under non-linear classification boundaries, the biases and MSEs of estimated overall and susceptible survival probabilities derived from the proposed MCM-MCIC-SVM model are the most minimal. Furthermore, these biases and MSEs exhibit a decreasing trend with an increase in sample size. These findings affirm that the proposed MCM-MCIC-SVM model’s adeptness in capturing non-linearity in the incidence also contributes to enhanced estimation results for the latency and overall survival probability. Table 5 displays the computation time (in seconds) required to obtain estimation results for a single dataset, incorporating both multiple imputation (with a size of 5) for the incidence and the bootstrap method (with a size of 100) for standard error estimation. Examining the outcomes in Table 5, it becomes evident that while our proposed methodologies entail considerable computational demands, it remains feasible to achieve estimation results within a reasonable timeframe. For interested readers, we also present in Table 6 the individual estimates and standard errors (based on 100 bootstrap samples) of the latency regression parameters for $n=300$. Note that in this case the true latency distribution is not the same as the fitted latency distribution since the baseline survival is estimated non-parametrically using the Turnbull method. For $n=600$, the observations are similar and not reported for the sake of brevity.

**Table 3:** Comparison of biases and MSEs of the overall survival probability for different models

Scenario	n	Bias			MSE		
		SVM	Spline	Logit	SVM	Spline	Logit
1	300	0.0065	0.0069	-0.0007	0.0112	0.0103	0.0097
	600	0.0014	0.0040	-0.0001	0.0091	0.0082	0.0081
2	300	0.0536	0.0770	0.0779	0.0250	0.0486	0.0565
	600	0.0503	0.0596	0.0685	0.0212	0.0445	0.0525
3	300	0.0217	0.0402	0.0291	0.0533	0.0562	0.0613
	600	0.0206	0.0337	0.0227	0.0515	0.0556	0.0588
4	300	0.0019	-0.0029	-0.0038	0.0689	0.0716	0.0721
	600	0.0016	-0.0021	-0.0026	0.0618	0.0659	0.0692

In the Supplementary Material, we present additional simulation results based on a data-driven approach to setting the parameter $\gamma$ and an extended range of *Q* values. Based on the results, higher values of *Q* are generally necessary for the SVM to achieve optimal performance.

**Table 4:** Comparison of biases and MSEs of the susceptible survival probability for different models

Scenario	n	Bias			MSE		
		SVM	Spline	Logit	SVM	Spline	Logit
1	300	0.0846	0.0876	0.0837	0.0607	0.0658	0.0542
	600	0.0826	0.0860	0.0817	0.0552	0.0553	0.0518
2	300	0.1061	0.1316	0.2007	0.0465	0.0814	0.1966
	600	0.1035	0.1305	0.1751	0.0462	0.0787	0.1663
3	300	0.1358	0.1363	0.1411	0.0689	0.0910	0.0749
	600	0.1255	0.1300	0.1367	0.0604	0.0815	0.0613
4	300	0.1074	0.1083	0.1086	0.0716	0.1029	0.1278
	600	0.0821	0.0830	0.1083	0.0421	0.0549	0.0807

## Analysis of HDSD data

The dataset comprises information on 238 subjects. The time to event is defined as the time to onset of grade IV VGE, which is interval censored. Covariates of interest include age, sex, TR360, and NOADYN. Age, measured in years, exhibits a mean of 31.882, with a standard deviation of 7.126, spanning from 20 to 54 years. Within the dataset, there are 177 males and 61 females. TR360, representing decompression stress, denotes the ratio of the partial pressure of nitrogen to ambient pressure at the final altitude. It has a mean of 1.637, a standard deviation of 0.227, and ranges from 1.04 to 1.89. NOADYN serves as an experimentally manipulated variable, indicating whether the test subject was ambulatory (NOADYN = 1) or experienced lower body adynamic conditions (NOADYN = 0) during the test session. Notably, 195 subjects exhibit NOADYN = 1. For further insights into the HDSD data, interested readers may refer to Conkin et al. (1992). We employ the proposed MCM-MCIC-SVM model to analyze the HDSD data. As noted by Ma (2010), only individual characteristics can influence susceptibility to grade IV VGE. Therefore, we set $\boldsymbol{z}=$ (Age, Sex). All four covariates are incorporated into the latency. Thus, we have $\boldsymbol{x}=$ (Age, Sex, TR360, NOADYN). The unknown model parameters are estimated using the EM algorithm outlined in Section 3. Standard errors of the estimates are computed using the bootstrap approach, based on 100 bootstrap samples. Additionally, for comparison purposes, we fit the MCM-MCIC-Logit and MCM-MCIC-Spline models to the HDSD data.

**Table 5:** Computation times for different models

Scenario	Model	Computation Time (in seconds)	
		*n*=300	*n*=600
1	SVM	269.75	793.53
	Spline	350.07	1028.76
	Logit	200.24	644.94
2	SVM	247.14	521.31
	Spline	356.95	809.55
	Logit	198.74	486.23
3	SVM	1126.91	1371.13
	Spline	1393.11	1842.01
	Logit	431.63	789.10
4	SVM	936.76	1456.05
	Spline	1107.14	2240.09
	Logit	511.26	1029.81

**Table 6:** Estimation of the latency regression parameters for $n=300$

Scenario	Model		Parameter									
			$\beta _1$	$\beta _2$	$\beta _3$	$\beta _4$	$\beta _5$	$\beta _6$	$\beta _7$	$\beta _8$	$\beta _9$	$\beta _{10}$
1	SVM	Est.	-4.812	4.825	-	-	-	-	-	-	-	-
		Std.	0.129	0.122	-	-	-	-	-	-	-	-
	Spline	Est.	-4.737	4.701	-	-	-	-	-	-	-	-
		Std.	0.147	0.161	-	-	-	-	-	-	-	-
	Logit	Est.	-3.346	4.013	-	-	-	-	-	-	-	-
		Std.	0.193	0.231	-	-	-	-	-	-	-	-
2	SVM	Est.	-4.728	4.037	-	-	-	-	-	-	-	-
		Std.	0.147	0.153	-	-	-	-	-	-	-	-
	Spline	Est.	-5.851	4.024	-	-	-	-	-	-	-	-
		Std.	0.180	0.189	-	-	-	-	-	-	-	-
	Logit	Est.	-3.010	3.099	-	-	-	-	-	-	-	-
		Std.	0.264	0.279	-	-	-	-	-	-	-	-
3	SVM	Est.	-1.230	1.622	0.286	1.534	-0.427	-0.830	-0.348	-1.009	0.809	1.396
		Std.	0.282	0.293	0.229	0.208	0.253	0.318	0.234	0.312	0.224	0.297
	Spline	Est.	-1.561	2.599	-0.482	1.880	-0.566	-0.624	-0.138	-1.329	0.932	0.987
		Std.	0.321	0.542	0.638	0.279	0.197	0.391	0.335	0.397	0.275	0.316
	Logit	Est.	0.203	0.643	-0.932	0.836	-0.017	-0.335	0.232	-0.546	0.242	0.377
		Std.	0.591	0.332	0.452	0.284	0.389	0.403	0.540	0.263	0.439	0.316
4	SVM	Est.	-0.822	1.452	0.745	1.419	-0.518	-1.570	-0.511	-0.876	0.125	1.843
		Std.	0.205	0.277	0.220	0.181	0.186	0.209	0.168	0.187	0.421	0.140
	Spline	Est.	-0.834	1.430	0.138	1.862	-0.509	-1.297	-0.487	-1.091	-0.163	1.897
		Std.	0.297	0.284	0.484	0.442	0.224	0.221	0.173	0.231	0.612	0.181
	Logit	Est.	-0.688	1.679	0.171	2.382	-0.515	-1.271	-0.361	-0.906	-0.125	1.731
		Std.	0.324	0.312	0.433	0.686	0.193	0.235	0.214	0.191	0.597	0.180

**Fig. 4:**
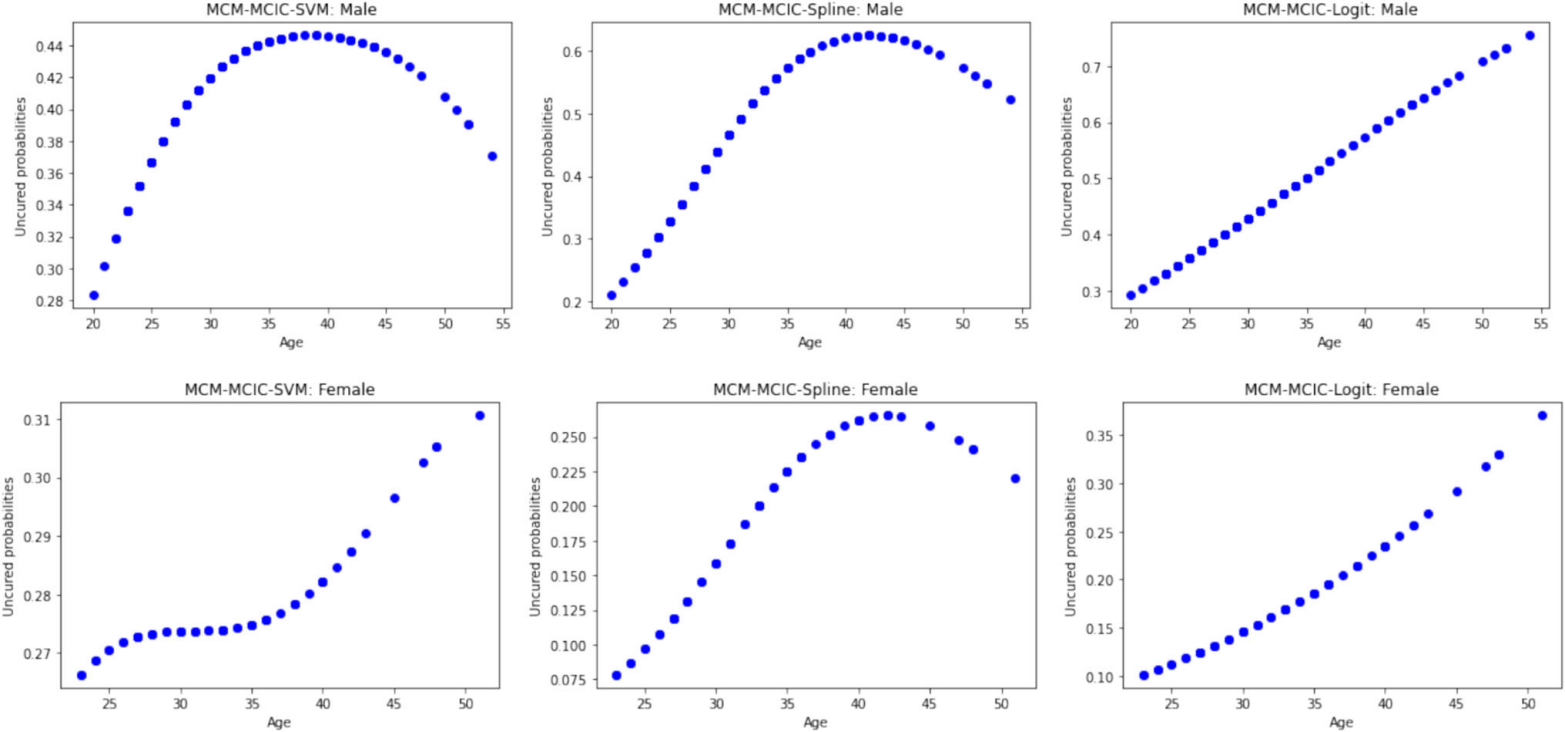
Estimated uncured probabilities as a function of age and sex for the HDSD data

The HDSD dataset exhibits significant imbalance, with 71% of patients being censored. Given that SVM-based models can be sensitive to such class imbalances, we employed a resampling technique to mitigate this issue during training. To address concerns about potential overfitting and contamination between training and testing, we initially split the HDSD data into two sets: 70% for training and 30% for testing. We then applied oversampling exclusively to the minority class within the training set, randomly augmenting or duplicating existing instances to achieve a more balanced class distribution, as suggested by Menardi and Torelli (2014). It is important to note that unlike some other techniques, oversampling ensures that all data instances are preserved, preventing loss of information during the model training process. The resultant training data was used to train the proposed model, whose performance was subsequently evaluated using the unseen test data.

**Fig. 5:**
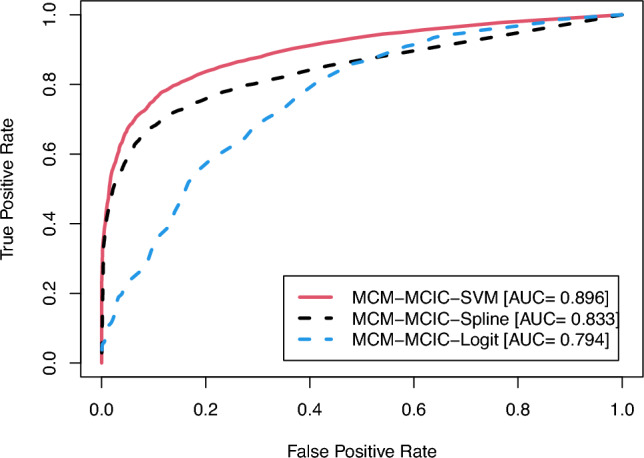
ROC curves and the corresponding AUC values for different models corresponding to the HDSD data

**Table 7:** Estimation results corresponding to the latency parameters for the HDSD data

Parameter	Estimates			SE			*p*-value		
	SVM	Spline	Logit	SVM	Spline	Logit	SVM	Spline	Logit
Age ($\beta _1$)	-0.1359	-0.2684	-0.2807	0.1221	0.1375	0.1328	0.0920	0.0509	0.0345
Sex ($\beta _2$)	0.0410	0.0005	-0.0060	0.1690	0.2401	0.2028	0.5085	0.7982	0.7769
TR360 ($\beta _3$)	-0.1058	-0.4227	-0.4175	0.4541	0.4682	0.4146	0.6157	0.3667	0.3140
Noadyn ($\beta _4$)	1.5871	1.8321	1.8272	0.4039	0.3969	0.3557	8.51$\times 10^{-05}$	3.92$\times 10^{-06}$	2.79$\times 10^{-07}$

Focusing on the inference concerning the incidence aspect, Figure 4 illustrates the estimates of uncured probabilities as a function of patients’ age and sex. The MCM-MCIC-SVM model adeptly captures the complex age effect on uncured probability, a capability also shared by the MCM-MCIC-Spline model but not by the MCM-MCIC-Logit model. Note that Ma (2010) highlighted a monotonic age effect on susceptibility to grade IV VGE, unlike our proposed MCM-MCIC-SVM model. On the other hand, males are more susceptible to grade IV VGE, a finding consistent with Ma (2010). Now, it is crucial to ascertain whether capturing this complex age effect translates into enhanced predictive accuracy for cure. To assess the predictive accuracies of all competing models using ROC curves and AUC values, we address missing cured statuses for censored lifetimes. Initially, we estimate the conditional probability of being uncured using eqn.(17) and the estimated values of $\pi (\boldsymbol{z})$ and $\boldsymbol{\beta }$ for each censored observation. Subsequently, based on these conditional uncured probabilities, we generate random variables from a Bernoulli distribution, representing cured/uncured statuses. This process is repeated 500 times, and the averaged ROC curves and AUC values are presented in Figure 5. It is worth noting that our proposed simulation-based approach for estimating missing cured statuses for ROC and AUC calculations is preferable as it avoids relying on simplistic assumptions such as the presence of a known “cured time", beyond which all censored observations are deemed cured (Asano et al. 2014). Clearly, the proposed MCM-MCIC-SVM model demonstrates the highest predictive accuracy for cure. Consequently, even in real-world scenarios where the true classification boundary is unknown, our model’s ability to capture complex age effects leads to enhanced predictive accuracy for cure. Precise estimation of cure rates is crucial because, in general, patients with significantly high cure rates can be shielded from the additional risks associated with intensive treatments. Likewise, patients with low cure rates can be promptly recommended treatment to prevent disease progression to stages where therapeutic options are limited.

Finally, our focus shifts to the inference pertaining to the latency component. In Table 7, we provide the estimations of the latency regression parameters along with their corresponding standard errors (SE) and *p*-values. Notably, at a significance level of 10%, only age and noadyn emerge as significant factors across all models concerning the time to onset of grade IV VGE for uncured patients. Additionally, the effects of age and noadyn remain consistent across all models. Specifically, younger patients and/or those who are ambulatory (NOADYN=1) exhibit a faster onset of grade IV VGE.

## Conclusion and future work

The SVM has garnered significant attention over the past two decades, proving its efficacy across various domains such as face detection, text categorization, and pedestrian detection. However, its application within the realm of cure rate models remains relatively novel and underexplored. In this study, we introduce a novel cure rate model that leverages SVM to model the incidence component and adopts a proportional hazards structure to address the latency aspect for time-to-event data subject to mixed case interval censoring. This innovative model inherits SVM’s capabilities, allowing it to capture more intricate classification boundaries. To estimate model parameters, we propose an EM algorithm employing sequential minimal optimization in conjunction with the Platt scaling method to estimate uncured probabilities. Given the unavailability of cured statuses for right-censored observations, we employ a multiple imputation-based approach to generate missing cured statuses. Due to the complexity of our model and estimation technique, we approximate standard errors of estimated parameters using a bootstrap approach. In our simulation study, we consider both simple scenarios involving two covariates and complex scenarios featuring ten covariates with correlated covariates and complicated interaction terms. Across all scenarios, our results demonstrate that when the true classification boundary is nonlinear, our proposed MCM-MCIC-SVM model excels in capturing nonlinearities within the data compared to the MCM-MCIC-Logit and MCM-MCIC-Spline models. This enhanced capability improves the accuracy and precision of uncured probability estimates. Additionally, we illustrate that our model’s adeptness at capturing nonlinear relationships leads to improved estimation results concerning overall and susceptible survival probabilities. Furthermore, our proposed model yields noticeably higher predictive accuracies compared to logit-based and spline-based models. These findings unequivocally highlight the superiority of our proposed model.

As a direct continuation of this research, there is significant interest in examining the performance of the proposed model in scenarios where the dimensionality of covariates is high, i.e., the sample size is smaller than the number of covariates. Additionally, there is a need to develop computationally efficient methods for covariate selection and evaluate their effectiveness. While our current study primarily focuses on SVM-based modeling, there is potential to compare SVM with other machine learning algorithms such as neural networks, decision trees, random forests, and gradient boosting, among others. Another avenue for future research involves extending the current framework to accommodate latent competing risks, as explored by Pal and Aselisewine (2023) in the context of right censored data, and investigating the possibility of risk elimination or reduction following an initial stage of treatment (Pal and Balakrishnan 2017b, 2018, 2016). These are ongoing areas of investigation, and we anticipate reporting our findings in forthcoming publications.

## Supplementary Information

Below is the link to the electronic supplementary material.

Supplementary file 1 (pdf 103 KB)

## Data Availability

Computational codes are available at https://github.com/Aselisewine/SVM-BASED-CURE-MODEL-MIXED-CASE-DATA.
