# Supplementary material for: A Support vector machine-based mixture cure model for mixed case interval censored data
Source: Stat Comput. 2026 Jan 16;36(2):63. doi: 10.1007/s11222-025-10796-3 (PMC12811344; doi:10.1007/s11222-025-10796-3)
Supplement: Supplementary file 1 — (pdf 103 KB) [file 11222_2025_10796_MOESM1_ESM.pdf]

Supplement to "A Support Vector Machine-Based Mixture Cure Model for Mixed Case Interval Censored Data"

Suvra Pal[1,2,*] **and Wisdom Aselisewine**[1]

[1]Department of Mathematics, University of Texas at Arlington, Texas, USA 76019
[2]Division of Data Science, College of Science, University of Texas at Arlington

1. Data-driven approach to γ and $Q = \{2^4, 2^5, 2^6\}$

Table 1: Comparison of AUC values for different models

Scenario	n	Training AUC			Testing AUC		
		SVM	Spline	Logit	SVM	Spline	Logit
	300	0.8672	0.8424	0.9683	0.8267	0.8442	0.9654
1	600	0.8384	0.8551	0.9693	0.7854	0.8517	0.9688
	300	0.8022	0.5464	0.5345	0.7260	0.5445	0.5452
2	600	0.7587	0.5410	0.5242	0.7135	0.5372	0.5274
	300	0.8038	0.6479	0.5728	0.7466	0.5701	0.5543
3	600	0.8664	0.6293	0.5668	0.7228	0.6194	0.5647
	300	0.8824	0.6891	0.5772	0.6720	0.5895	0.5553
4	600	0.9142	0.6694	0.5785	0.6970	0.6055	0.5547

*Corresponding author. E-mail address: suvra.pal@uta.edu Tel.:817 272 7163

Table 2: Comparison of biases and MSEs of the uncured probability for different models

Scenario	n	Bias			MSE		
		SVM	Spline	Logit	SVM	Spline	Logit
	300	-0.0344	-0.1221	0.0362	0.1250	0.1140	0.0185
1	600	-0.0107	-0.1125	0.0334	0.0925	0.0969	0.0128
	300	-0.0227	0.1246	0.1308	0.0550	0.1154	0.2186
2	600	-0.0118	0.1136	0.1083	0.0502	0.1140	0.2028
	300	0.0288	0.1344	0.1642	0.1053	0.1933	0.1637
3	600	0.0170	0.1268	0.1592	0.0909	0.1724	0.1566
	300	0.0535	0.0837	0.1532	0.1070	0.1536	0.1980
4	600	-0.0157	0.0819	0.1271	0.1054	0.1268	0.1633

Table 3: Comparison of biases and MSEs of the overall survival probability for different models

Scenario	n	Bias			MSE		
		SVM	Spline	Logit	SVM	Spline	Logit
	300	0.0255	0.0069	-0.0007	0.0266	0.0103	0.0097
1	600	0.0201	0.0040	-0.0001	0.0200	0.0082	0.0081
	300	0.0645	0.0770	0.0779	0.0463	0.0486	0.0565
2	600	0.0636	0.0596	0.0685	0.0441	0.0445	0.0525
	300	0.0432	0.0402	0.0291	0.0448	0.0562	0.0613
3	600	0.0289	0.0337	0.0227	0.0421	0.0556	0.0588
	300	-0.0024	-0.0029	-0.0038	0.0693	0.0716	0.0721
4	600	-0.0019	-0.0021	-0.0026	0.0638	0.0659	0.0692

Table 4: Comparison of biases and MSEs of the susceptible survival probability for different models

Scenario	n	Bias			MSE		
		SVM	Spline	Logit	SVM	Spline	Logit
	300	0.1103	0.0876	0.0837	0.0974	0.0658	0.0542
1	600	0.0644	0.0860	0.0817	0.0516	0.0553	0.0518
	300	0.1118	0.1316	0.2007	0.0538	0.0814	0.1966
2	600	0.0922	0.1305	0.1751	0.0372	0.0787	0.1663
	300	0.1217	0.1363	0.1411	0.0757	0.0910	0.0749
3	600	0.1180	0.1300	0.1367	0.0513	0.0815	0.0613
	300	0.0982	0.1083	0.1086	0.0552	0.1029	0.1278
4	600	0.0812	0.0830	0.1083	0.0502	0.0549	0.0807

2. Data-driven approach to γ and $Q = \{2^{-3}, 2^{-2}, 2^{-1}, 2^{0}, 2^{1}, \cdots, 2^{6}\}$

Table 5: Comparison of AUC values for different models

Scenario	n	Training AUC			Testing AUC		
		SVM	Spline	Logit	SVM	Spline	Logit
1	300	0.8673	0.8424	0.9683	0.8344	0.8442	0.9654
2	300	0.8107	0.5464	0.5345	0.7198	0.5445	0.5452
3	300	0.8100	0.6479	0.5728	0.7543	0.5701	0.5543
4	300	0.8765	0.6891	0.5772	0.6722	0.5895	0.5553

Table 6: Comparison of biases and MSEs of the uncured probability for different models

		Bias			MSE		
Scenario	n	SVM	Spline	Logit	SVM	Spline	Logit
1	300	-0.0417	-0.1221	0.0362	0.1101	0.1140	0.0185
2	300	-0.0231	0.1246	0.1308	0.0550	0.1154	0.2186
3	300	0.0287	0.1344	0.1642	0.0987	0.1933	0.1637
4	300	0.0525	0.0837	0.1532	0.1042	0.1536	0.1980

3. Data-driven approach to γ and $Q = \{2^{-3}, 2^{-2}, 2^{-1}, 2^{0}, 2^{1}, 2^{2}, 2^{3}\}$

Table 7: Comparison of AUC values for different models

Scenario	n	Training AUC			Testing AUC		
		SVM	Spline	Logit	SVM	Spline	Logit
1	300	0.8621	0.8424	0.9683	0.8504	0.8442	0.9654
2	300	0.7511	0.5464	0.5345	0.5608	0.5445	0.5452
3	300	0.7702	0.6479	0.5728	0.6426	0.5701	0.5543
4	300	0.5421	0.6891	0.5772	0.4796	0.5895	0.5553

Table 8: Comparison of biases and MSEs of the uncured probability for different models

		Bias			MSE		
Scenario	n	SVM	Spline	Logit	SVM	Spline	Logit
1	300	-0.0604	-0.1221	0.0362	0.0884	0.1140	0.0185
2	300	-0.0482	0.1246	0.1308	0.0604	0.1154	0.2186
3	300	0.0170	0.1344	0.1642	0.0949	0.1933	0.1637
4	300	0.4113	0.0837	0.1532	0.2838	0.1536	0.1980
